# Supplementary material for: RNA and Protein Determinants Mediate Differential Binding of miRNAs by a Viral Suppressor of RNA Silencing Thus Modulating Antiviral Immune Responses in Plants
Source: Int J Mol Sci. 2022 Apr 29;23(9):4977. doi: 10.3390/ijms23094977 (PMC9103804; doi:10.3390/ijms23094977)
Supplement: Supplementary file 1 [file ijms-23-04977-s001.zip › ijms-1700465-supplementary.pdf]

SUPPLEMENTARY MATERIAL

Supplementary Table S1: List of DNA oligonucleotides.

Name	Sequence (5’-3’)	Purpose
p19pGEXf	GCGGATCCATGGAACGAGCTAT ACAAGGAAAC	PCR primer for *CIRV* p19 cloning (forward)
p19pGEXr	TCCCCCGGGGAATTACTCGCTT TCTTTCTTGAAGGTTTC	PCR primer for *CIRV* p19 cloning (reverse)
pSUMOTAV2b1f	GACCATGAAGACCGTGGTATG GCAAGCATCGAGATCCCTC	PCR primer for *TAV* 2b cloning (forward)
pSUMOTAV2b1r	CCGCTCGAGTTATTGATCGAGA CAC	PCR primer for *TAV* 2bcloning (reverse)
p19Q6Afwd	GCCATGGAACGAGCTATAgcAG GAAACGACACTAGGG	forward primer for site-directed mutagenesis of CIRV p19 (Q6A)
p19Q6Arev	CCCTAGTGTCGTTTCCTgcTATA GCTCGTTCCATGGC	reverse primer for site-directed mutagenesis of CIRV p19 (Q6A)
p19N8Afwd	GGAACGAGCTATACAAGGAgcC GACACTAGGGAACAAGC	forward primer for site-directed mutagenesis of CIRV p19 (N8A)
p19N8Arev	GCTTGTTCCCTAGTGTCGgcTC CTTGTATAGCTCGTTCC	reverse primer for site-directed mutagenesis of CIRV p19 (N8A)
p19K67Afwd	GCTGGGGTTTCGGGgcAGTTGT ATTTAAGAGATATCTCAG	forward primer for site-directed mutagenesis of CIRV p19 (K67A)
p19K67Arev	CTGAGATATCTCTTAAATACAAC TgcCCCGAAACCCCAGC	reverse primer for site-directed mutagenesis of CIRV p19 (K67A)
p19N106Afwd	CGATTTCTCGGTGCCgcCCAGG TCGGATGTACC	forward primer for site-directed mutagenesis of CIRV p19 (N106A)
p19N106Arev	GGTACATCCGACCTGGgcGGCA CCGAGAAATCG	reverse primer for site-directed mutagenesis of CIRV p19 (N106A)
p19Q107Afwd	CGATTTCTCGGTGCCAACgctGT CGGATGTACCTATAGTATTCG	forward primer for site-directed mutagenesis of CIRV p19 (Q107A)
p19Q107Arev	CGAATACTATAGGTACATCCGA CagcGTTGGCACCGAGAAATCG	reverse primer for site-directed mutagenesis of CIRV p19 (Q107A)

p19V108Afwd	CTCGGTGCCAACCAGGcCGGAT GTACCTATAGTATTCGG	forward primer for site-directed mutagenesis of CIRV p19 (V108A)
p19V108Arev	CCGAATACTATAGGTACATCCG gCCTGGTTGGCACCGAG	reverse primer for site-directed mutagenesis of CIRV p19 (V108A)
p19G109Afwd	CGGTGCCAACCAGGTCGcATGT ACCTATAGTATTCGGTTTCG	forward primer for site-directed mutagenesis of CIRV p19 (G109A)
p19G109Arev	CGAAACCGAATACTATAGGTAC ATgCGACCTGGTTGGCACCG	reverse primer for site-directed mutagenesis of CIRV p19 (G109A)
p19T111Afwd	CAACCAGGTCGGATGTgCCTAT AGTATTCGGTTTCGAG	forward primer for site-directed mutagenesis of CIRV p19 (T111A)
p19T111Arev	CTCGAAACCGAATACTATAGGc ACATCCGACCTGGTTG	reverse primer for site-directed mutagenesis of CIRV p19 (T111A)

Lower case letters indicate nucleotide exchanges to be implemented in the coding sequence of p19.

Supplementary Table S2: List of RNA oligonucleotides.

Name	Sequence (5'-3')	Purpose
gf698 gs	uag uuc auc cau gcc aug ugu	guide strand of gf698 siRNA
gf698 ps	aca ugg cau gga uga acu aua	passenger strand of gf698 siRNA
*At*miR162+	ucg aua aac cuc ugc auc cag	guide strand of *At*miR162 (isoforms a and b) and *Nb*miR162 (isoform a)
*At*miR162*	gga ggc agc ggu uca ucg auc	passenger strand of *At*miR162 (isoforms a and b) and *Nb*miR162 (isoform a)
*Nb*miR162b*	gga ggc agc ggu uua ucg auc	passenger strand of *Nb*miR162 (isoform b)
miR162+-10u	ucg aua aac uuc ugc auc cag	modified guide strand of *At*miR162 carrying the mutation C10U
miR162+-12g	ucg aua aac cug ugc auc cag	modified guide strand of *At*miR162 carrying the mutation C12G
miR162+-12u	ucg aua aac cuu ugc auc cag	modified guide strand of *At*miR162 carrying the mutation C12U
miR162+-13g	ucg aua aac cuc ggc auc cag	modified guide strand of *At*miR162 carrying the mutation U13G
miR162+-14u	ucg aua aac cuc uuc auc cag	modified guide strand of *At*miR162 carrying the mutation G14U
miR162+-15u	ucg aua aac cuc ugu auc cag	modified guide strand of *At*miR162 carrying the mutation C15U
miR162+-16u	ucg aua aac cuc ugc uuc cag	modified guide strand of *At*miR162 carrying the mutation A16U
miR162+-18u	ucg aua aac cuc ugc auu cag	modified guide strand of *At*miR162 carrying the mutation C18U
miR162+-19u	ucg aua aac cuc ugc auc uag	modified guide strand of *At*miR162 carrying the mutation C19U
miR162*-0	gga ugc aga ggu uca ucg auc	modified passenger strand of *At*miR162 carrying mutations G4U and C9A
miR162*-1c	cga ugc aga ggu uca ucg auc	modified version of miR162*-0 carrying the mutation G1C

miR162*-2c	gca ugc aga ggu uca ucg auc	modified version of miR162*-0 carrying the mutation G2C
miR162*-3c	ggc ugc aga ggu uca ucg auc	modified version of miR162*-0 carrying the mutation A3C
miR162*-3g	ggg ugc aga ggu uca ucg auc	modified version of miR162*-0 carrying the mutation A3G
miR162*-4g	gga ggc aga ggu uca ucg auc	modified version of miR162*-0 carrying the mutation U4G
miR162*-5c	gga ucc aga ggu uca ucg auc	modified version of miR162*-0 carrying the mutation G5C
miR162*-6a	gga uga aga ggu uca ucg auc	modified version of miR162*-0 carrying the mutation C6A
miR162*-6g	gga ugg aga ggu uca ucg auc	modified version of miR162*-0 carrying the mutation C6G
miR162*-6u	gga ugu aga ggu uca ucg auc	modified version of miR162*-0 carrying the mutation C6U
miR162*-7c	gga ugc cga ggu uca ucg auc	modified version of miR162*-0 carrying the mutation A7C
miR162*-7g	gga ugc gga ggu uca ucg auc	modified version of miR162*-0 carrying the mutation A7G
miR162*-7u	gga ugc uga ggu uca ucg auc	modified version of miR162*-0 carrying the mutation A7U
miR162*-8a	gga ugc aaa ggu uca ucg auc	modified version of miR162*-0 carrying the mutation G8A
miR162*-8c	gga ugc aca ggu uca ucg auc	modified version of miR162*-0 carrying the mutation G8C
miR162*-8u	gga ugc aua ggu uca ucg auc	modified version of miR162*-0 carrying the mutation G8U
miR162*-9c	gga ugc agc ggu uca ucg auc	modified version of miR162*-0 carrying the mutation A9C
miR162*-9g	gga ugc agg ggu uca ucg auc	modified version of miR162*-0 carrying the mutation A9G

miR162*-10c	gga ugc aga cgu uca ucg auc	modified version of miR162*-0 carrying the mutation G10C
*At*miR168+	ucg cuu ggu gca ggu cgg gaa	guide strand of *At*miR168 (isoforms a and b) and *Nb*miR168 (isoforms d and e)
*At*miR168a*	ccc gcc uug cau caa cug aau	passenger strand of *At*miR168 (isoform a) and *Nb*miR168 (all isoforms)
*At*miR168b*	ccc guc uug uau caa cug aau	passenger strand of *At*miR168 (isoform b)
*Nb*miR168a,b,c+	ucg cuu ggu gca ggu cgg gac	guide strand of *Nb*miR168 (isoforms a,b and c)
miR168*V1	ccc gcc cug cau caa cug aau	modified version of *At*miR168a* carrying the mutation U7C
miR168*V2	ccc gcc uug cac caa cug aau	modified version of *At*miR168a* carrying the mutation U12C
miR168*V3	ccc gcc cug cac caa cug aau	modified version of *At*miR168a* carrying the mutations U7C and U12C
miR168*V4	ccc gac cug cau caa aug aau	modified version of *At*miR168a* carrying the mutations C5A, U7C and C16G
miR168*V5	ccc gac uug cac caa gug aau	modified version of *At*miR168a* carrying the mutations C5A, U12C and C16G
miR168*V6	ccc gac cug cac caa gcg aau	modified version of *At*miR168a* carrying the mutations C5A,U7C, U12C, C16G and U17C
*At*miR403+	uua gau uca cgc aca aac ucg	guide strand of *At*miR403 and *Nb*miR403 (both isoforms)
*At*miR403*	ugu uuu gug cuu gaa ucu aau u	passenger strand of *At*miR403
*Nb*miR403a*	ugu uug ugc gug aau cug aca	passenger strand of *Nb*miR403 (isoform a)
*Nb*miR403b*	ugu uug ugc gug auu cug aca	passenger strand of *Nb*miR403 (isoform b)

Supplementary Table S3: Duplex schemes of non-mutated sRNAs.

<table>
<tr><th>Name and duplex scheme</th><th>Description</th></tr>
<tr><td>gf698

5´-UAGUUCAUCCAUGCCAUGUGU-3´
|||||||||||||||||||
3´-AUAUCAAGUAGGUACGGUACA-5´</td><td>gf698 siRNA</td></tr>
<tr><td>AtmiR162a,b

5´-UCGAU^{A}AACC^{U}CUGC^{A}UCCAG-3´
||||| |||| |||| |||
3´-CUAGCUA$_{C}$UUGG$_{C}$GACG$_{G}$AGG-5´</td><td>miR162 from Arabidopsis thaliana, isoforms a and b</td></tr>
<tr><td>NbmiR162a

5´-UCGAUAAACC^{U}CUGC^{A}UCCAG-3´
|||||||||| |||| |||
3´-CUAGCUAUUUGG$_{C}$GACG$_{G}$AGG-5´</td><td>miR162 from Nicotiana benthamiana, isoform a</td></tr>
<tr><td>NbmiR162b

5´-UCGAU^{A}AACC^{U}CUGC^{A}UCCAG-3´
||||| |||| |||| |||
3´-CUAGCUA$_{C}$UUGG$_{C}$GACG$_{G}$AGG-5´</td><td>miR162 from Nicotiana benthamiana, isoform b</td></tr>
<tr><td>AtmiR168a

5´-UCG^{C}UUGGUGCAGG^{U}CGGGAA-3´
||• |||•||||•| ||||
3´-UAAGU$_{C}$AACUACGUUC$_{C}$GCCC-5´</td><td>miR168 from Arabidopsis thaliana, isoform a</td></tr>
<tr><td>AtmiR168b

5´-UCG^{C}UUGGUGCAGG^{U}CGGGAA-3´
||• |||•|•||•| ||||
3´-UAAGU$_{C}$AACUAUGUUC$_{C}$GCCC-5´</td><td>miR168 from Arabidopsis thaliana, isoform b</td></tr>
<tr><td>NbmiR168a,b,c

5´-UCG^{C}UUGGUGCAGG^{U}CGGGAC-3´
||• |||•||||•| ||||
3´-UAAGU$_{C}$AACUACGUUC$_{C}$GCCC-5´</td><td>miR168 from Nicotiana benthamiana, isoforms a, b and c</td></tr>
<tr><td>NbmiR168d,e

5´-UCG^{C}UUGGUGCAGG^{U}CGGGAA-3´
||• |||•||||•| ||||
3´-UAAGU$_{C}$AACUACGUUC$_{C}$GCCC-5´</td><td>miR168 from Nicotiana benthamiana, isoforms d and e</td></tr>
</table>

Supplementary Table S4: Duplex schemes of mutated miR162 variants.

<table>
<tr><th>Name and duplex scheme</th><th>Position of non-canonical base pair</th><th>Introduced base pair</th><th>RNA oligonucleotides annealed</th></tr>
<tr><td>miR162-0
5´-UCGAU^{A}AACCUCUGCAUCCAG-3´
||||| |||||||||||||
3´-CUAGCUA$_{C}$UUGGAGACGUAGG-5´</td><td>-</td><td>-</td><td>AtmiR162+
miR162*-0</td></tr>
<tr><td>miR162-1*C/19^{g}C
5´-UCGAU^{A}AACCUCUGCAUC^{C}AG-3´
||||| ||||||||||||
3´-CUAGCUA$_{C}$UUGGAGACGUAG$_{C}$-5´</td><td>1*/19^{g}</td><td>^{g}-strand: C
*-strand: C</td><td>AtmiR162+
miR162*-1c</td></tr>
<tr><td>miR162-2*C/18^{g}C
5´-UCGAU^{A}AACCUCUGCAU^{C}CAG-3´
||||| ||||||||||| |
3´-CUAGCUA$_{C}$UUGGAGACGUA$_{C}$G-5´</td><td>2*/18^{g}</td><td>^{g}-strand: C
*-strand: C</td><td>AtmiR162+
miR162*-2c</td></tr>
<tr><td>miR162-3*C/17^{g}U
5´-UCGAU^{A}AACCUCUGCA^{U}CCAG-3´
||||| |||||||||| ||
3´-CUAGCUA$_{C}$UUGGAGACGU$_{C}$GG-5´</td><td>3*/17^{g}</td><td>^{g}-strand: U
*-strand: C</td><td>AtmiR162+
miR162*-3c</td></tr>
<tr><td>miR162-4*G/16^{g}A
5´-UCGAU^{A}AACCUCUGC^{A}UCCAG-3´
||||| ||||||||| |||
3´-CUAGCUA$_{C}$UUGGAGACG$_{G}$AGG-5´</td><td>4*/16^{g}</td><td>^{g}-strand: A
*-strand: G</td><td>AtmiR162+
miR162*-4g</td></tr>
<tr><td>miR162-5*C/15^{g}C
5´-UCGAU^{A}AACCUCUG^{C}AUCCAG-3´
||||| |||||||| ||||
3´-CUAGCUA$_{C}$UUGGAGAC$_{C}$UAGG-5´</td><td>5*/15^{g}</td><td>^{g}-strand: C
*-strand: C</td><td>AtmiR162+
miR162*-5c</td></tr>
<tr><td>miR162-6*A/14^{g}G
5´-UCGAU^{A}AACCUCU^{G}CAUCCAG-3´
||||| ||||||| |||||
3´-CUAGCUA$_{C}$UUGGAGA$_{A}$GUAGG-5´</td><td>6*/14^{g}</td><td>^{g}-strand: G
*-strand: A</td><td>AtmiR162+
miR162*-6a</td></tr>
<tr><td>miR162-7*C/13^{g}U
5´-UCGAU^{A}AACCUC^{U}GCAUCCAG-3´
||||| |||||| ||||||
3´-CUAGCUA$_{C}$UUGGAG$_{C}$CGUAGG-5´</td><td>7*/13^{g}</td><td>^{g}-strand: U
*-strand: C</td><td>AtmiR162+
miR162*-7c</td></tr>
</table>

<table>
<tr><td>miR162-8*C/12^{g}C

5´-UCGAU^{A}AACCU^{C}UGCAUCCAG-3´
||||| ||||| |||||||
3´-CUAGCUA$_{C}$UUGGA$_{C}$ACGUAGG-5´</td><td>8*/12^{g}</td><td>^{g}-strand: C
*-strand: C</td><td>AtmiR162+
miR162*-8c</td></tr>
<tr><td>miR162-9*C/11^{g}U

5´-UCGAU^{A}AACC^{U}CUGCAUCCAG-3´
||||| |||| ||||||||
3´-CUAGCUA$_{C}$UUGG$_{C}$GACGUAGG-5´</td><td>9*/11^{g}</td><td>^{g}-strand: U
*-strand: C</td><td>AtmiR162+
miR162*-9c</td></tr>
<tr><td>miR162-10*C/10^{g}C

5´-UCGAU^{A}AAC^{C}UCUGCAUCCAG-3´
||||| ||| |||||||||
3´-CUAGCUA$_{C}$UUG$_{C}$AGACGUAGG-5´</td><td>10*/10^{g}</td><td>^{g}-strand: C
*-strand: C</td><td>AtmiR162+
miR162*-10c</td></tr>
<tr><td>miR162-1*G/19^{g}U

5´-UCGAU^{A}AACCUCUGCAUCUAG-3´
||||| ||||||||||||•
3´-CUAGCUA$_{C}$UUGGAGACGUAGG-5´</td><td>1*/19^{g}</td><td>^{g}-strand: U
*-strand: G</td><td>miR162+-19u
miR162*-0</td></tr>
<tr><td>miR162-2*G/18^{g}U

5´-UCGAU^{A}AACCUCUGCAUUCAG-3´
||||| |||||||||||•|
3´-CUAGCUA$_{C}$UUGGAGACGUAGG-5´</td><td>2*/18^{g}</td><td>^{g}-strand: U
*-strand: G</td><td>miR162+-18u
miR162*-0</td></tr>
<tr><td>miR162-3*G/17^{g}U

5´-UCGAU^{A}AACCUCUGCAUCCAG-3´
||||| ||||||||||•||
3´-CUAGCUA$_{C}$UUGGAGACGUGGG-5´</td><td>3*/17^{g}</td><td>^{g}-strand: U
*-strand: G</td><td>AtmiR162+
miR162*-3g</td></tr>
<tr><td>miR162-4*G/16^{g}U

5´-UCGAU^{A}AACCUCUGCUUCCAG-3´
||||| |||||||||•|||
3´-CUAGCUA$_{C}$UUGGAGACGGAGG-5´</td><td>4*/16^{g}</td><td>^{g}-strand: U
*-strand: G</td><td>miR162+-16u
miR162*-4g</td></tr>
<tr><td>miR162-5*G/15^{g}U

5´-UCGAU^{A}AACCUCUGUAUCCAG-3´
||||| ||||||||•||||
3´-CUAGCUA$_{C}$UUGGAGACGUAGG-5´</td><td>5*/15^{g}</td><td>^{g}-strand: U
*-strand: G</td><td>miR162+-15u
miR162*-0</td></tr>
<tr><td>miR162-6*G/14^{g}U

5´-UCGAU^{A}AACCUCUUCAUCCAG-3´
||||| |||||||•|||||
3´-CUAGCUA$_{C}$UUGGAGAGGUAGG-5´</td><td>6*/14^{g}</td><td>^{g}-strand: U
*-strand: G</td><td>miR162+-14u
miR162*-6g</td></tr>
</table>

miR162-7*G/13^{g}U<br>5´-UCGAU^{A}AACCUCUGCAUCCAG-3´<br>||||| ||||||•|||||| 3´-CUAGCUA$_{C}$UUGGAGGCGUAGG-5´	7*/13^{g}	^{g}-strand: U<br>*-strand: G	*At*miR162+<br>miR162*-7g
miR162-8*G/12^{g}U<br>5´-UCGAU^{A}AACCUUUGCAUCCAG-3´<br>||||| |||||•||||||| 3´-CUAGCUA$_{C}$UUGGAGACGUAGG-5´	8*/12^{g}	^{g}-strand: U<br>*-strand: G	miR162+-12u<br>miR162*-0
miR162-9*G/11^{g}U<br>5´-UCGAU^{A}AACCUCUGCAUCCAG-3´<br>||||| ||||•|||||||| 3´-CUAGCUA$_{C}$UUGGGGACGUAGG-5´	9*/11^{g}	^{g}-strand: U<br>*-strand: G	*At*miR162+<br>miR162*-9g
miR162-10*G/10^{g}U<br>5´-UCGAU^{A}AACUUCUGCAUCCAG-3´<br>||||| |||•||||||||| 3´-CUAGCUA$_{C}$UUGGAGACGUAGG-5´	10*/10^{g}	^{g}-strand: U<br>*-strand: G	miR162+-10u<br>miR162*-0
miR162-6*U/14^{g}G<br>5´-UCGAU^{A}AACCUCUGCAUCCAG-3´<br>||||| |||||||•||||| 3´-CUAGCUA$_{C}$UUGGAGAUGUAGG-5´	6*/14^{g}	^{g}-strand: G<br>*-strand: U	*At*miR162+<br>miR162*-6u
miR162-7*U/13^{g}G<br>5´-UCGAU^{A}AACCUCGGCAUCCAG-3´<br>||||| ||||||•|||||| 3´-CUAGCUA$_{C}$UUGGAGUCGUAGG-5´	7*/13^{g}	^{g}-strand: G<br>*-strand: U	miR162+-13g<br>miR162*-7u
miR162-8*G/12^{g}G<br>5´-UCGAU^{A}AACCU^{G}UGCAUCCAG-3´<br>||||| ||||| ||||||| 3´-CUAGCUA$_{C}$UUGGA$_{G}$ACGUAGG-5´	8*/12^{g}	^{g}-strand: G<br>*-strand: G	miR162+-12g<br>miR162*-0
miR162-8*A/12^{g}C<br>5´-UCGAU^{A}AACCU^{C}UGCAUCCAG-3´<br>||||| ||||| ||||||| 3´-CUAGCUA$_{C}$UUGGA$_{A}$ACGUAGG-5´	8*/12^{g}	^{g}-strand: C<br>*-strand: A	*At*miR162+<br>miR162*-8a
miR162-8*U/12^{g}G<br>5´-UCGAU^{A}AACCUGUGCAUCCAG-3´<br>||||| |||||•||||||| 3´-CUAGCUA$_{C}$UUGGAUACGUAGG-5´	8*/12^{g}	^{g}-strand: G<br>*-strand: U	miR162+-12g<br>miR162*-8u

Changes in the sequence in comparison to *At*miR162 wt (for miR162-0) or to miR162-0 are indicated in blue. The right column lists RNA oligonucleotides that where annealed to form the corresponding duplexes. For ssRNA sequence information, see Table S2.

Supplementary Table S5: Duplex schemes of mutated miR168 variants.

<table>
<tr><th>Name and duplex scheme</th><th>Description</th><th>RNA oligonucleotides annealed</th></tr>
<tr><td>AtmiR168a variant 1

5´-UCG^CUUGGUGCAGG^UCGGGAA-3´
||• |||•|||||| ||||
3´-UAAGU_CAACUACGUCC_CGCCC-5´</td><td>variant 1 of miR168 from Arabidopsis thaliana</td><td>AtmiR168+ miR168*V1</td></tr>
<tr><td>AtmiR168a variant 2

5´-UCG^CUUGGUGCAGG^UCGGGAA-3´
||• ||||||||•| ||||
3´-UAAGU_CAACCACGUUC_CGCCC-5´</td><td>variant 2 of miR168 from Arabidopsis thaliana</td><td>AtmiR168+ miR168*V2</td></tr>
<tr><td>AtmiR168a variant 3

5´-UCG^CUUGGUGCAGG^UCGGGAA-3´
||• |||||||||| ||||
3´-UAAGU_CAACCACGUCC_CGCCC-5´</td><td>variant 3 of miR168 from Arabidopsis thaliana</td><td>AtmiR168+ miR168*V3</td></tr>
<tr><td>AtmiR168a variant 4

5´-UCGCUUGGUGCAGGUCGGGAA-3´
||•||||•|||||||||||
3´-UAAGUGAACUACGUCCAGCCC-5´</td><td>variant 4 of miR168 from Arabidopsis thaliana</td><td>AtmiR168+ miR168*V4</td></tr>
<tr><td>AtmiR168a variant 5

5´-UCGCUUGGUGCAGGUCGGGAA-3´
||•|||||||||•||||||
3´-UAAGUGAACCACGUUCAGCCC-5´</td><td>variant 5 of miR168 from Arabidopsis thaliana</td><td>AtmiR168+ miR168*V5</td></tr>
<tr><td>AtmiR168a variant 6

5´-UCGCUUGGUGCAGGUCGGGAA-3´
|||||||||||||||||||
3´-UAAGCGAACCACGUCCAGCCC-5´</td><td>variant 6 of miR168 from Arabidopsis thaliana</td><td>AtmiR168+ miR168*V6</td></tr>
</table>

Nucleotide exchanges in comparison to the wt miRNA are indicated in blue. The right column lists RNA oligonucleotides which where annealed to form the corresponding duplex. For ssRNA sequence information, see Table S2.

Supplementary Figure legends

Figure S1. *In vitro* translation of *CIRV* p19 wt and mutant proteins in BYL. *CIRV* p19 mutant RNAs were *in vitro* translated as described in Materials and Methods in the presence of L-[^{35}S]-methionine. (A) Autoradiography of a representative SDS-PAGE of the translated proteins. (B) Bands corresponding to the radiolabeled p19 variants were quantified and the relative band intensities normalized to the corresponding bands of the p19 wt protein. Bars represent means of three independent experiments with standard deviations. The experiments indicated no significant differences in the translation efficiencies of the different p19 variants.

Figure S2. Far-UV circular dichroism (CD) spectra of recombinantly produced and purified p19 variants. The spectra of p19 wt (black), p19 Q107A (cyan), p19 V108A (red) and p19 G109A (blue) display the same shape and the same ratio of the signal intensities at 208 nm and 218 nm. The overall signal amplitude of p19 G109A is about half of that of the wild type, indicating some structural perturbation as a result of the mutation (change in asymmetry/chirality).

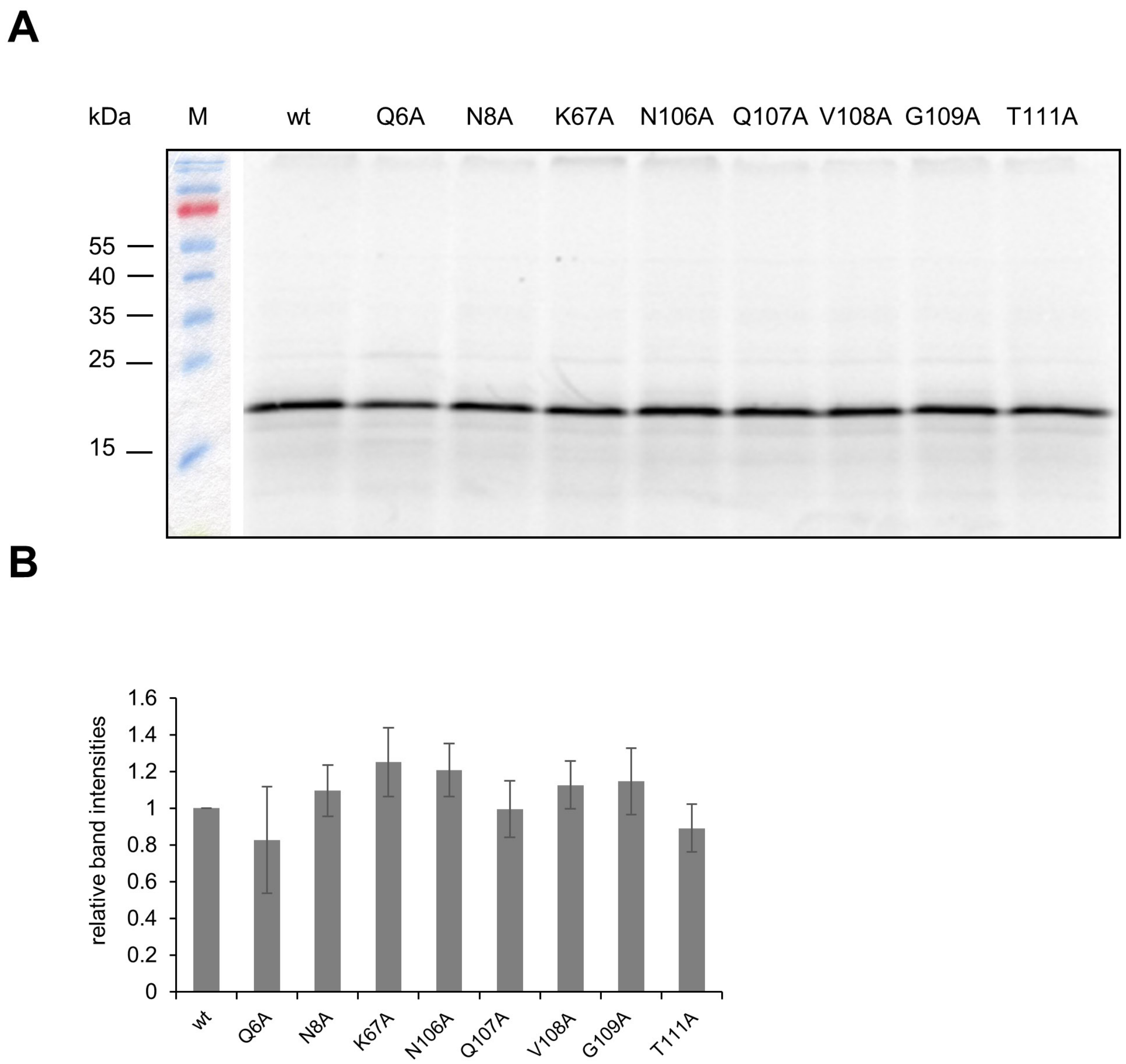
A
kDa
M
wt
Q6A
N8A
K67A
N106A
Q107A
V108A
G109A
T111A
55
40
35
25
15
B
relative band intensities
1.6
1.4
1.2
1
0.8
0.6
0.4
0.2
0
wt
Q6A
N8A
K67A
N106A
Q107A
V108A
G109A
T111A

Figure S1

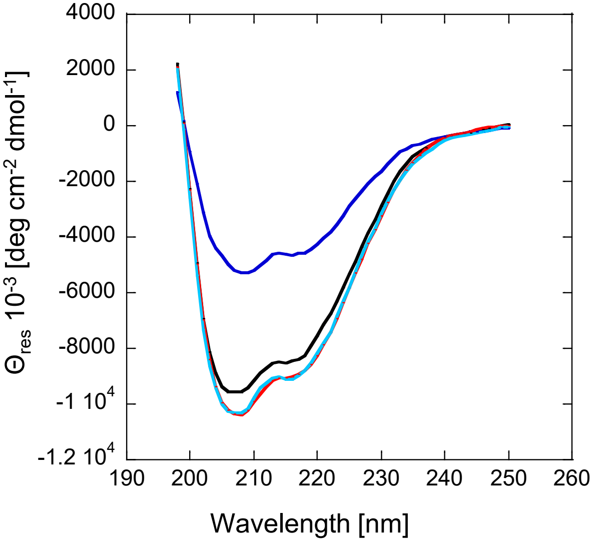
4000
2000
0
-2000
-4000
-6000
-8000
-1 10^4
-1.2 10^4
190
200
210
220
230
240
250
260
Wavelength [nm]
Θres 10-3 [deg cm-2 dmol-1]

Figure S2
